# Active and passive smoking and the risk of myocardial infarction in 24,968 men and women during 11 year of follow-up: the Tromsø Study

**DOI:** 10.1007/s10654-013-9785-z

**Published:** 2013-02-27

**Authors:** Birgitte Iversen, Bjarne K. Jacobsen, Maja-Lisa Løchen

**Affiliations:** Department of Community Medicine, Faculty of Health Sciences, University of Tromsø, 9037 Tromsø, Norway

**Keywords:** Cigarette smoking, Involuntary smoking, Passive smoking, Sex, Myocardial infarction

## Abstract

Active smoking is a well-established risk factor for myocardial infarction, but less is known about the impact of passive smoking, and possible sex differences in risk related to passive smoking. We investigated active and passive smoking as risk factors for myocardial infarction in an 11-year follow-up of 11,762 men and 13,206 women included in the Tromsø Study. There were a total of 769 and 453 incident cases of myocardial infarction in men and women, respectively. We found linear age-adjusted relationships between both active and passive smoking and myocardial infarction incidence in both sexes. The relationships seem to be stronger for women than for men. Age-adjusted analyses indicated a stronger relationship with passive smoking in ever-smokers than in never-smokers. After adjustment for important confounders (body mass index, blood pressure, total cholesterol, HDL cholesterol and physical activity) the associations with active and passive smoking were still statistically significant. Adjusting for active smoking when assessing the effect of passive smoking and vice versa, indicated that the effect of passive smoking in men may be explained by their own active smoking. In women, living with a smoker ≥30 years after the age of 20 increased the myocardial infarction risk by 40 %, even after adjusting for active smoking. Passive smoking is a risk factor for myocardial infarction on its own, but whereas the effect for men seems to be explained by their own active smoking, the effect in females remains statistically significant.

## Introduction

Smoking is the most important preventable risk factor for disease and premature death. Even though the smoking prevalence in Norway has nearly been halved during the last 15 years, 17 % of Norwegian men and women aged 16–74 were daily smokers in 2011 [1] and the prevalence does not differ much between men and women. The risks of smoking in relation to myocardial infarction (MI) are well-known [2], but several studies have found that the impact of smoking is larger in women than in men. A recently published large meta-analysis of prospective cohort studies [3] found that female smokers had 25 % higher adjusted risk of coronary heart disease than men who smoke. The higher risk of women compared to men may for different reasons be underestimated [3], and a previous study from Norway indicated an approximately adjusted 75 % higher risk in female than in male smokers [4].

Passive smoking (second hand tobacco smoke), has been shown to increase the risk of coronary heart disease [5–8]. One prospective study with objective measurement of exposure found no relationship, however, but living with a smoker was associated with a two-fold increased myocardial infarction risk in non-smoking participants [9].

There has, however, been little research on whether the observed sex difference in risk related to active smoking also applies for passive smoking. A meta-analysis from 1999 indicated no difference between the genders [6]. A recent case–control study from Costa Rica showed that the risk of myocardial infarction increased by 23 % in subjects living with a smoker and the strength of the relationship did not differ much between the genders [8]. Thus, the aim of this population-based prospective study was to elucidate active and passive smoking as risk factors for myocardial infarction with emphasis on possible sex differences.

## Methods

### The Tromsø Study

The Tromsø Study is a population-based prospective study established in 1974. There have been a total of six surveys in the Tromsø Study, the last in 2007–2008. The present study is based on data from the Tromsø 4 survey. This is the largest survey conducted as a part of the Tromsø Study and took place during 1994–1995. All residents in the municipality of Tromsø born before 1970 (i.e. aged 25 and above) were invited to participate and 27,158 (77 %) attended [10]. The participants received a questionnaire along with the invitation and were asked to bring the completed form when they came for the physical examination. The questionnaire included questions concerning, among other topics, active and passive smoking as well as physical activity.

### Measurements and questionnaires

Height and weight measurements were performed in light clothing. The participant was taken into a separate room with only one nurse present to measure the blood pressure. The blood pressure was measured three times after the participant had been seated for 2 min using an automatic device (Dinamap Vital Signs Monitor 1846; Criticon, Inc, Tampa, FL). The mean of the two last measurements was used in the present analyses.

To obtain the blood sample a venipuncture was performed while the person was seated. The blood test included measurement of serum total cholesterol and HDL cholesterol. Analysis of serum total cholesterol was done by enzymatic colorimetric methods with commercial kits (CHOD-PAP; Boehringer-Manheim, Mannheim, Germany). Serum high-density lipoprotein (HDL) was measured after precipitation of lower density lipoproteins with manganese chloride. All the analyses were performed by the Department of Clinical Chemistry, University Hospital of North Norway.

Subjects were defined as physically inactive if they reported never participating in hard leisure activities that would make them sweat or be out of breath, in addition to being lightly active less than 1 h per week. The participants were defined as physically moderate active if they reported light leisure activity for more than 1 h per week, and some, although less than 1 h per week, hard leisure activity. Participants reporting hard leisure activity for more than 1 h per week were considered physically active.

Information about history of active smoking was based on several questions in the questionnaire. With regard to active smoking, the subjects were categorized into seven different categories: Never smokers, ex-smokers who quit ≥20 years ago, who quit 10–19 years ago, who quit less than 10 years ago, current smoker who smokes less than 10 cigarettes per day, who smoke 10–19 cigarettes per day and who currently smoke ≥20 cigarettes per day.

There were two questions concerning passive smoking and we assessed passive smoking as two different variables. The first was based on a question in the questionnaire asking for how many years the participants had been living with a daily smoker after their 20th birthday. Their exposure to passive smoking at home was then categorized in five different groups: Never exposed, exposed less than 10 years, exposed 10–19 years, exposed 20–29 years and exposed for more than 30 years. The second variable was related to how many hours per day the respondent was present in smoke-filled rooms. They answered by giving an estimate in hours, in our analyses categorized into three groups (0, 1–6 and >6 h per day). The two indicators of exposure to cigarette smoke were statistically significantly (*p* < 0.001), but not highly, correlated (Spearman correlation = 0.31 and 0.26 in men and women, respectively).

### Identification of myocardial infarction cases and follow-up

Hospitalized cases of incident MI were identified by linking the Tromsø Study participant list to the discharge diagnosis register at the University Hospital of North Norway as described elsewhere [11]. This hospital is the only hospital in the area. To identify all possible first-ever MI cases, our search strategy included the following diagnostic codes: from 1980 to 1998 ICD 9 codes 410-414 and 798-7998; and thereafter ICD 10 codes I20-I25, and R96 and R98-99. The hospital medical record was then retrieved for case validation. Discharge letters from hospitalizations in other hospitals were also collected when appropriate. Further, the Tromsø Study participant list was linked with the nationwide Causes of Death Registry at Statistics Norway and the death certificates were retrieved for those with an underlying or contributing diagnosis of CVD or sudden unexpected death. Relevant information was collected from additional sources such as autopsy reports and records from nursing homes, ambulance services and general practitioners. This procedure identified fatal incident cases of MI that occurred as out-of-hospital deaths, including deaths that occurred outside Tromsø. Cases meeting diagnostic criteria for definite or probable fatal or non-fatal first-ever MI were classified as MI. WHO MONICA/MORGAM criteria were used in the algorithms and included clinical symptoms and signs, findings in electrocardiograms, values of cardiac biomarkers and (when applicable) autopsy reports. Silent MIs as defined by ECG only were not included as cases because of difficulties in determining the exact date of the event. In the present analyses, we included MI endpoints till the end of 2005.

### Analytical sample

A total of 26,957 persons attended the Tromsø 4 survey and gave informed consent. Data from 24,968 men and women were included in the present analyses. Subjects excluded had experienced a myocardial infarction prior to the physical examination (n = 684) or had missing values regarding information about active and passive smoking, physical activity or the physical examination (n = 1,305).

### Statistical analysis

Cox regression was performed to analyze active and passive smoking as risk factors for myocardial infarction during follow-up. First we only adjusted for age at start of follow-up, then we adjusted in concert for age, mean systolic blood pressure, total serum cholesterol, serum HDL, body mass index (BMI, kg/m^2^) and physical activity and finally, for active smoking (for passive smoking) and passive smoking (for active smoking). The analysis was performed separately for men and women. Stratified analyses were performed in order to investigate whether the risk from passive smoking differed between ever-smokers and never smokers.

The study follow-up started the day the participant were clinically examined and lasted till the day they had their first myocardial infarction, died, emigrated or till the end of follow-up (end of 2005), whichever came first.

Ninety-five percent confidence intervals were calculated. Two-sided *p* values <0.05 were considered statistically significant. All analyses were performed using SPSS 16.0 software (SPSS Inc. Chicago, Illinois, USA).

## Results

### Characteristics of cases of incident MI

During follow-up, 3.4 % of the females and 6.5 % of the males had an incident myocardial infarction. There were a total of 1,222 cases of myocardial infarction, 769 cases in men and 453 in women. Age at start of follow-up was higher (68.0 years) in female cases of myocardial infarction than in male cases (60.7 years). Age at enrollment in subjects who did not have a myocardial infarction during follow-up was, as expected, considerably lower than in subjects who had a myocardial infarction during follow-up and did not differ much between women (45.8 years) and men (44.7 years). The unadjusted baseline characteristics are presented in Table 1. Gender specific analyses indicated no statistically significant (*p* ≥ 0.6) linear relationships between time spent in smoke-filled rooms and myocardial infarction incidence. In women, no significant relationship was found for active smoking (*p* = 0.12).

**Table 1:** Baseline characteristics in men and women, according to myocardial infarction (MI) during follow-up. The Tromsø Study 1994–1995

	Men (n = 11,762)		Women (n = 13,206)	
	No MI (N = 10,993)	MI (N = 769)	No MI (N = 12,753)	MI (N = 453)
Age	44.7 (13.5)	60.7 (12.8)	45.8 (14.8)	68.0 (11.7)
BMI (kg/m^2^)	25.5 (3.3)	26.6 (3.6)	24.7 (4.2)	26.8 (4.8)
Serum lipids (mmol/l)				
Total cholesterol	5.96 (1.20)	6.82 (1.17)	5.97 (1.36)	7.28 (1.35)
HDL	1.35 (0.35)	1.31 (0.35)	1.65 (0.40)	1.58 (0.44)
Blood pressure (mm Hg)				
Systolic	136.3 (16.4)	149.4 (21.6)	130.8 (21.5)	161.6 (26.5)
Diastolic	79.4 (11.5)	86.9 (13.2)	75.8 (12.3)	88.5 (15.5)
Physical activity (%)				
Inactive	16.7	24.2	19.2	39.3
Moderate	44.1	51.0	55.8	51.2
Active	39.2	24.8	25.0	9.5
Smoking habits (%)				
Never-smoker	32.3	15.0	39.8	46.6
Stopped ≥20 years ago	9.0	19.6	5.1	5.7
Stopped 10–19 years ago	7.9	10.9	7.1	3.5
Stopped <10 years ago	13.4	12.1	12.1	5.5
Current <10 cigarettes/day	7.6	8.8	11.7	15.5
Current 10–19 cigarettes/day	19.5	21.2	20.0	18.3
Current ≥20 cigarettes/day	10.3	12.4	4.3	4.9
Living with a smoker^a^ (%)				
Never	33.3	24.7	30.8	17.0
<10 years	21.4	7.3	20.6	3.3
10–19 years	18.3	13.3	19.1	10.4
20–29 years	13.9	18.6	14.1	17.2
≥30 years	13.0	36.2	15.4	52.1
Time spent in smoke-filled rooms (%)				
0 h/day	41.7	48.9	55.9	59.4
1–6 h/day	39.8	27.2	29.5	23.2
>6 h/day	18.4	23.9	14.6	17.4

Values are mean (standard deviations in parentheses) or percentages

^a^ If a person currently is or has been living with a smoker since his/her 20th birthday

Subjects who suffered a myocardial infarction during follow-up had higher body mass index, blood pressure and total cholesterol and lower HDL cholesterol and less physical activity than subjects who did not have a myocardial infarction. All of these differences were statistically significant (*p* ≤ 0.002).

### Active and passive smoking

Overall, we found that 36 % of the women and 38 % of the men were current smokers (data not shown). Men did, however, smoke more than did the females. Whereas 1,227 (10.4 %) of the 11,762 males smoked more than 20 cigarettes per day, only 565 (4.3 %) of the 13,206 females did so (Table 2). There was not much difference between men and women with regard to for how long time they had been living with a smoker; approximately 15 % reported to have lived with a smoker for more than 30 years. Men reported more often than women to spend much time in smoke-filled rooms (Table 2).

**Table 2:** Active and passive smoking as risk factors for myocardial infarction (MI) in men and women, The Tromsø Study, 1994–2005

	Men (n = 11,762)					Women (n = 13,206)				
	N^a^	n^b^	HR^c^ (95 % CI)	HR^d^ (95 % CI)	HR^e^ (95 % CI)	N	n	HR^c^ (95 % CI)	HR^d^ (95 % CI)	HR^e^ (95 % CI)
Own smoking habits										
Never-smoker	3,668	115	1 (–)	1 (–)	1 (–)	5,285	211	1 (–)	1 (–)	1 (–)
Stopped ≥20 years ago	1,141	151	1.3 (1.0–1.6)	1.2 (1.0–1.6)	1.2 (0.9–1.5)	672	26	0.8 (0.5–1.1)	0.8 (0.5–1.2)	0.8 (0.5–1.2)
Stopped 10–19 years ago	947	84	1.5 (1.1–2.0)	1.3 (1.0–1.8)	1.3 (0.9–1.7)	919	16	0.8 (0.5–1.3)	0.9 (0.5–1.4)	0.8 (0.5–1.4)
Stopped <10 years ago	1,569	93	1.4 (1.1–1.9)	1.3 (1.0–1.7)	1.2 (0.9–1.6)	1,572	25	1.2 (0.8–1.9)	1.3 (0.9–2.0)	1.2 (0.8–1.8)
Current <10 cigarettes/day	898	68	1.5 (1.1–2.1)	1.6 (1.2–2.2)	1.5 (1.1–2.0)	1,560	70	2.3 (1.8–3.1)	2.6 (1.9–3.4)	2.2 (1.6–3.0)
Current 10–19 cigarettes/day	2,312	163	1.9 (1.5–2.4)	1.8 (1.4–2.3)	1.7 (1.3–2.2)	2,633	83	2.5 (1.9–3.2)	2.7 (2.0–3.5)	2.2 (1.6–3.1)
Current ≥20 cigarettes/day	1,227	95	2.5 (1.9–3.3)	2.2 (1.7–2.9)	1.9 (1.4–2.6)	565	22	3.5 (2.2–5.6)	3.6 (2.3–5.7)	3.1 (1.8–5.1)
*p* value linear trend			<0.001	<0.001	<0.001			<0.001	<0.001	<0.001
Living with a smoker^f^										
Never	3,853	190	1 (–)	1 (–)	1 (–)	4,011	77	1 (–)	1 (–)	1 (–)
<10 years	2,412	56	0.8 (0.6–1.1)	0.9 (0.7–1.2)	0.8 (0.6–1.1)	2,643	15	0.6 (0.4–1.1)	0.7 (0.4–1.2)	0.7 (0.4–1.2)
10–19 years	2,119	102	1.2 (1.0–1.6)	1.2 (0.9–1.5)	1.1 (0.8–1.4)	2,477	47	1.2 (0.8–1.7)	1.3 (0.9–1.8)	1.2 (0.8–1.7)
20–29 years	1,674	143	1.5 (1.2–1.9)	1.4 (1.1–1.7)	1.2 (1.0–1.5)	1,872	78	1.5 (1.1–2.1)	1.5 (1.1–2.1)	1.3 (1.0–1.9)
≥30 years	1,704	278	1.5 (1.2–1.8)	1.3 (1.1–1.6)	1.1 (0.9–1.4)	2,203	236	1.8 (1.4–2.3)	1.8 (1.4–2.3)	1.4 (1.1–1.9)
*p* value linear trend			<0.001	< 0.001	0.145			<0.001	<0.001	0.004
Time spent in smoke-filled rooms										
0 h/day	4,965	376	1 (–)	1 (–)	1 (–)	7,392	269	1 (–)	1 (–)	1 (–)
1–6 h/day	4,589	209	1.1 (0.9–1.3)	1.1 (1.0–1.4)	1.0 (0.8–1.2)	3,873	105	1.8 (1.5–2.3)	1.9 (1.5–2.3)	1.2 (0.9–1.6)
>6 h/day	2,208	184	1.7 (1.4–2.0)	1.6 (1.3–1.9)	1.2 (0.9–1.4)	1,941	79	2.1 (1.6–2.7)	2.0 (1.6–2.7)	1.1 (0.8–1.5)
*p* value linear trend			<0.001	<0.001	0.198			<0.001	<0.001	0.182

^a^ N Number of subjects included in the analyses at baseline

^b^ n Number of cases of myocardial infarction

^c^ Hazard ratios (95 % confidence interval), adjusted for age

^d^ Adjusted for age, mean systolic blood pressure, total serum cholesterol, serum HDL, BMI and physical activity

^e^ Adjusted for age, mean systolic blood pressure, total serum cholesterol, serum HDL, BMI and physical activity as well as passive smoking (for active smoking) or active smoking (for passive smoking)

^f^ If a person currently is or has been living with a smoker since his/her 20th birthday

Table 2 shows active and passive smoking as risk factors for myocardial infarction. Age-adjusted analyses (first column of hazard ratios) demonstrate that the risk of myocardial infarction increased linearly for both active and passive smoking in both sexes, but rather consistently stronger for women than for men. Formal test for interaction by sex for the three variables were not statistically significant or only borderline significant, however. After adjustments for important confounders (body mass index, blood pressure, total cholesterol, HDL cholesterol and physical activity), the effects of active and passive smoking were still statistically significant (second column). Adjusting for active smoking when assessing the effect of passive smoking and vice versa (third column) indicated that the effect of passive smoking in men can be explained by their own active smoking, while in women the effect of passive smoking, operationalized by the number of years living with a smoker, still was statistically significantly associated with myocardial infarction incidence and indicated a 40 % increased risk of myocardial infarction in women who had lived with a smoker for ≥30 years. A formal test indicated a marginally statistically significant (*p* = 0.03) interaction by sex when all three interactions terms for the smoking variables were included in the model as a block. In a fully adjusted model (third column for each sex in Table 2) including in addition one interaction term (for “Own smoking habits”, “Living with a smoker”, or “Time spent in smoke filled rooms”) at the time demonstrated a significant interaction with sex for the two first variables (*p* = 0.02 and 0.04, respectively) and no significant interaction (*p* = 0.14) for the last variable.

### Passive smoking and incident MI in never and ever smokers

Passive smoking was a risk factor for myocardial infarction in never smoking women (Table 3). Both age-adjusted and adjusted for the other confounders there was in women a positive dose–response association between living with a smoker and the risk of myocardial infarction in females with a 60 % (95 % CI 10–120) increased risk in women living with a smoker for more than 29 years compared with a female who never had been living with smoker after the age of 20. There was no effect of living with a smoker in men and no effect of time spent in a smoke-filled room in neither of the sexes.

**Table 3:** Passive smoking as a risk factor for myocardial infarction (MI) in men and women who have never been actively smoking, The Tromsø Study, 1994–2005

	Men (n = 3,668)					Women (n = 5,285)				
	N^a^	N^b^	HR^c^ (95 % CI)	HR^d^ (95 % CI)	HR^e^ (95 % CI)	N^a^	N^b^	HR^c^ (95 % CI)	HR^d^ (95 % CI)	HR^e^ (95 % CI)
Living with smoker^f^										
Never	2,226	68	1 (–)	1 (–)	1 (–)	2,529	49	1 (–)	1 (–)	1 (–)
< 10 years	759	10	0.7 (0.4–1.4)	0.7 (0.4–1.4)	0.7 (0.4–1.5)	803	8	0.9 (0.4–1.8)	0.9 (0.4–2.0)	1.0 (0.5–2.1)
10–19 years	364	13	1.2 (0.6–2.1)	1.2 (0.6–2.1)	1.2 (0.6–2.1)	583	22	1.4 (0.8–2.3)	1.4 (0.8–2.2)	1.4 (0.8–2.3)
20–29 years	194	8	0.8 (0.4–1.7)	0.7 (0.3–1.5)	0.7 (0.3–1.6)	528	31	1.4 (0.9–2.2)	1.3 (0.8–2.1)	1.3 (0.9–2.1)
≥30 years	125	16	1.2 (0.7–2.1)	1.1 (0.6–2.0)	1.1 (0.6–2.1)	842	101	1.4 (1.0–2.0)	1.5 (1.0–2.1)	1.6 (1.1–2.2)
*p* value linear trend			0.731	0.959	0.885			0.028	0.021	0.009
Time spent in smoke-filled rooms										
0 h/day	2,272	86	1 (–)	1 (–)	1 (–)	4,220	189	1 (–)	1 (–)	1 (–)
1–6 h/day	1,231	26	1.1 (0.7–1.7)	1.0 (0.6–1.6)	1.0 (0.6–1.6)	866	15	0.8 (0.5–1.3)	0.7 (0.5–1.3)	0.7 (0.4–1.2)
>6 h/day	165	3	0.7 (0.2–2.3)	0.8 (0.3–2.6)	0.8 (0.3–2.7)	199	7	0.9 (0.4–1.8)	0.8 (0.4–1.7)	0.7 (0.3–1.5)
*p* value linear trend			0.865	0.785	0.761			0.393	0.348	0.138

^a^ N Number of subjects included in the analyses at baseline

^b^ n Number of cases of myocardial infarction

^c^ Hazard ratios (95 % confidence interval), adjusted for age

^d^ Adjusted for age, mean systolic blood pressure, total serum cholesterol, serum HDL, BMI and physical activity

^e^ Adjusted for age, mean systolic blood pressure, total serum cholesterol, serum HDL, BMI and physical activity as well as living with a smoker (for smoke-filled room) and smoke-filled room (for living with smoker)

^f^ If a person currently is or has been living with a smoker since his/her 20^th^ birthday

Similarly, Table 4 shows passive smoking as a predictor for myocardial infarction in ever smoking subjects (current or previous smokers). In analyses only adjusted for age, we found an association for both variables conveying information about passive smoking and myocardial infarction risk in both sexes (albeit the relationship seems stronger in women). The effect of living with a smoker was attenuated when adjusting for the other cardiovascular risk factors, but it was still statistically significant. When adjusting for their own active smoking and the time spent in smoke filled rooms in addition, the effect of living with a smoker was still significant in women (HR = 1.3 for ≥30 years; 95 % CI 0.9–2.0; *p* value for linear trend = 0.04).

**Table 4:** Passive smoking as a risk factor for myocardial infarction (MI) in men and women who currently are or previously have been actively smoking, The Tromsø Study, 1994–2005

	Men (n = 8,094)					Women (n = 7,921)				
	N^a^	N^b^	HR^c^ (95 % CI)	HR^d^ (95 % CI)	HR^e^ (95 % CI)	N^a^	N^b^	HR^c^ (95 % CI)	HR^d^ (95 % CI)	HR^e^ (95 % CI)
Living with smoker^f^										
Never	1,627	122	1 (–)	1 (–)	1 (–)	1,482	28	1 (–)	1 (–)	1 (–)
<10 years	1,653	46	0.8 (0.5–1.1)	0.8 (0.6–1.2)	0.9 (0.6–1.2)	1,840	7	0.4 (0.2–0.9)	0.4 (0.2–1.0)	0.5 (0.2–1.1)
10–19 years	1,755	89	1.1 (0.8–1.4)	1.0 (0.8–1.4)	1.1 (0.8–1.4)	1,894	25	0.9 (0.5–1.6)	1.0 (0.6–1.7)	1.0 (0.6–1.7)
20–29 years	1,480	135	1.4 (1.1–1.8)	1.3 (1.0–1.6)	1.3 (1.0–1.6)	1,344	47	1.4 (0.9–2.2)	1.4 (0.9–2.2)	1.3 (0.8–2.1)
≥30 years	1,579	262	1.4 (1.1–1.7)	1.2 (1.0–1.5)	1.1 (0.9–1.4)	1,361	135	1.8 (1.2–2.7)	1.7 (1.1–2.6)	1.3 (0.9–2.0)
*p* value linear trend			<0.001	0.01	0.108			<0.001	<0.001	0.044
Time spent in smoke-filled rooms										
0 h/day	2,693	290	1 (–)	1 (–)	1 (–)	3,172	80	1 (–)	1 (–)	1 (–)
1–6 h/day	3,358	183	1.0 (0.8–1.2)	1.1 (0.9–1.4)	1.0 (0.8–1.2)	3,007	90	2.6 (1.9–3.5)	2.5 (1.8–3.4)	1.7 (1.2–2.4)
>6 h/day	2,043	181	1.5 (1.2–1.8)	1.5 (1.2–1.8)	1.2 (0.9–1.5)	1,742	72	2.6 (1.9–3.6)	2.4 (1.7–3.4)	1.4 (1.0–2.1)
*p* value linear trend			<0.001	<0.001	0.132			<0.001	<0.001	0.084

^a^ N Number of subjects included in the analyses at baseline

^b^ n Number of cases of myocardial infarction

^c^ Hazard ratios (95 % confidence interval), adjusted for age

^d^ Adjusted for age, mean systolic blood pressure, total serum cholesterol, serum HDL, BMI and physical activity

^e^ Adjusted for age, mean systolic blood pressure, total serum cholesterol, serum HDL, BMI and physical activity as well as active smoking, living with a smoker (for smoke-filled room) and smoke-filled room (for living with smoker)

^f^ If a person currently is or has been living with a smoker since his/her 20th birthday

### Stronger relationships in younger subjects

Age-stratified analyses suggest a stronger impact of both active and passive smoking in the relatively young compared to older subjects. A formal test indicated a statistically significant (*p* = 0.004 and 0.03 in men and women, respectively) linear interaction with age when all three interaction terms for the smoking variables were included in the model as a block. In a fully adjusted model (third column for each sex in Table 2) including in addition one interaction term (for “Own smoking habits”, “Living with a smoker”, or “Time spent in smoke filled rooms”) at the time demonstrated a significant interaction with age for all three variables for men (*p* ≤ 0.004) and for “Living with a smoker” in women (*p* = 0.004). The effects of smoking were strongest for “Own smoking habits” and in women. Younger and middle-aged females, aged 25–54 at enrollment (69 cases of myocardial infarction), who currently smoked ≥20 cigarettes per day had a higher risk of myocardial infarction (HR = 3.8; 95 % CI 1.4–9.9) than females aged 55-69 (HR = 2.2; 95 % CI 1.0–4.9) and females aged ≥70 years (HR = 1.6; 95 % CI 0.4–6.9). The same analysis for men showed lower estimates; HR = 2.7 (95 % CI 1.7–4.3) in men aged 25–54 at enrollment, HR = 1.1 (95 % CI 0.7–1.9) in men aged 55–69 and HR = 1.0 (95 % CI 0.3–3.0) in men aged ≥70 years at enrollment. One should note the wide confidence intervals, however.

### Population attributable risk for passive smoking

Applying the adjusted risk estimates in women who never had been a smoker, we found that the population attributable risk (PAR) for myocardial infarction for ever been exposed to passive smoking (i.e., ever lived a smoker since her 20th birthday) was 19 %, while it was 18 % in all women (data not shown). As we only found a significant relationship between passive smoking and myocardial infarction risk in women, we only report the PAR estimate for women.

## Discussion

The present study confirms that smoking increases the incidence of myocardial infarction and supports the notion that the relationships are stronger in women than in men [2–4]. Furthermore, this prospective cohort study found that passive smoking, at least in women, was an independent risk factor for myocardial infarction. The age-adjusted relationship in women between years living with a smoker after the age of 20 and myocardial infarction risk was as least as strong in ever smokers as in never smokers. In men, most of the association with passive smoking was probably explained by confounders, particularly their own active smoking.

### Comparison with previous studies and explanations for the findings

Previous studies concerning the impact of passive smoking on myocardial infarction risk have shown that passive smoking or living with a smoker increases the risk of myocardial infarction [5–9]. Our results (at least before adjustments for active smoking were undertaken) are in accordance with this. Furthermore, we find that the effect of passive smoking was more pronounced in women. The latter seems to be in variance with most previous studies [6, 8, 9].

The findings of the large INTERHART case–control study indicated that the effect of passive smoking was weaker in current than in ex- or never smokers [7], but other studies have not found major differences [5, 8, 9].

We have no obvious explanation for the probably stronger relationship in women for both active and passive smoking with regard to the risk of myocardial infarction, and not much is known about how smoking may have different impacts in men and women [12]. It is possible that there is an interaction between some female specific risk factors (e.g., polycystic ovarian syndrome (PCOS), the use of oral contraceptives) and smoking. PCOS affects 5–10 % of women in a general population [13, 14] and is associated with several known risk factors for myocardial infarction (e.g., obesity, insulin resistance). The use of oral contraceptives in combination with smoking increases the risk of myocardial infarction [12], but this is of marginal relevance in this study as there were few cases among the females in fertile age. Some studies have shown an inverse relationship between age at menopause and myocardial infarction risk [15], and smoking lowers age at natural menopause by 1–2 years [16]. This could contribute to the higher myocardial infarction rate in female smokers. One likely explanation for the fact that the association of passive smoking with MI incidence was found predominately in women may be that men tend to smoke more than women. In current smokers, 28 and 12 % of men and women, respectively, smoked 20 cigarettes or more per day. Hence, a woman living with a male smoker might have a higher exposure to passive smoking than a man living with a female smoker.

Our data also support that the impact of smoking (both active and passive) on the MI incidence is stronger in younger than in older subjects. This was not unexpected as the relative risk associated with smoking is higher in relatively young individuals than in older subjects [2].

The INTERHEART study showed that in subjects who never had smoked cigarettes, passive smoking accounted for 10.8 and 18.6 % of all myocardial infarctions in women and men, respectively [7]. Another study indicated that passive smoking accounted for 9 % of the female cases and 7 % of the male cases [8]. The present study found that the PAR for passive smoking was 18 % in women, but it is difficult to compare PAR estimates from previous studies because of different threshold for being considered exposed for passive smoking as well as different prevalence of passive smoking.

### Limitations and strengths

Population studies like this one may be prone to selection bias, as there will be the people who are healthy that are able to come to the clinical examination and participate in the study. We have previously reported lower mortality in attendees to the Tromsø 4 survey according to attendance to previous surveys they were invited to [10] and similar studies conducted in Norway have demonstrated higher morbidity and mortality in non-attenders than in subjects who took part in the surveys [17, 18]. The higher morbidity in non-attenders does, however, influence our findings only if the relationship between smoking and myocardial infarction is different in the large majority (77 %) who chose to attend the Tromsø 4 survey and in those who did not attend.

Data concerning smoking habits in the study were from a self-administered questionnaire which may have introduced an information bias. Some previous studies have used cotinine measurements as an objective measure of passive smoking [5, 9], only one of them demonstrated an association between serum cotinine level and myocardial infarction risk [5]. We have no objective measures of tobacco exposure like cotinine or thiocyanate. A previous Norwegian study showed that the relation between self-reported smoking habits and the measure of serum thiocyanate was strong if the question was asked in a neutral setting [19]. As the questions about active and passive smoking were included in a self-administered questionnaire, in a neutral setting, we believe that the validity was good, although we recognize that the tobacco use stated in this study probably is underreported.

In the present study we used two variables to measure passive smoking: How long they had been living with a smoker after their 20th birthday and how many hours per day they have spent in a smoke-filled room. The two variables were not highly correlated (Spearman correlation was approximately 0.3). Thus, the two variables probably reflect different types of exposure. We found that the relationships with myocardial infarction were stronger with years lived with a smoker than with number of hours per day in smoke-filled rooms. It is possible that the “smoke-filled room” question is somewhat ambiguous as it may include smoke filled rooms at workplaces (due to ambient air pollution) as well as passive smoking at home and at the workplace. This ambiguity might constitute a possible information bias, and we believe that it is correct to rather focus on the “living with smoker” variable which clearly reflects exposure to cigarette smoking.

Another possible limitation is the fact that the survey was administered in 1994, and we have no information regarding changes in smoking habits, both active and passive smoking, during follow-up. As the smoking prevalence in the Norwegian population has been reduced, we assume that some subjects classified as “current smokers” were ex-smokers by the time they had their infarction. Thus, our estimates of the risk of myocardial infarction associated with current smoking are probably underestimated because some in the “current smokers” group truly were “ex-smokers”, hence contributing a lower risk than a true “current smoker” to the results. Similar effects may have taken place with regard to exposure to passive smoking although the information concerning long duration of living with a smoker has probably not changed much during follow-up. It is a common finding in prospective studies that the relationship between the exposure and end-point becomes weaker with increasing duration of follow-up. This has also been seen in a study of the effect of passive smoking [5].

The study has, however, also some important strengths. This population based prospective study included 24,968 participants and the participation rate to the survey was high (77 %). The analyses are prospective, including incident cases, which rules out the effect of disease on the reporting of smoking habits. The analyses include all fatal and non-fatal cases in the study population, not only the non-fatal cases as in case–control studies [7, 8]. Out of the 411 men who died during follow-up, 194 (47 %) died within 2 days of the MI, i.e., most probably due to the MI. The corresponding figure in women was 50 % (144 of 289 deaths). Thus, nearly 30 % of the cases would have not been available for interview in a case–control study. Furthermore, we were able to adjust for baseline levels of the other traditional risk factors for myocardial infarction (total cholesterol, HDL cholesterol, blood pressure and physical activity), and we have also adjusted for active smoking when assessing the effect of passive smoking and vice versa.

## Conclusion

The present study confirmed that smoking is an important risk factor for myocardial infarction and that smoking also in this respect is more detrimental for women [3]. The results for passive smoking give furthermore some support for a stronger effect in women than men. Passive smoking is a risk factor for myocardial infarction, but whereas the effect for men seems to be explained by their own active smoking, the effect in females remains statistically significant and approximately 20 % of the incident cases of myocardial infarction in women in this population can be attributed to passive smoking. Thus, the public health impact of our findings is not negligible. More research is, however, needed into the mechanisms that contribute to this elevated risk, particularly with regard to the possible sex differences.
